# Foreign Summary

**Published:** 1838-05-23

**Authors:** 


### FOREIGN SUMMARY.

*M. Ricord's Practice in Gonorrhœa.*—During the acute stage of gonorrhœa, when the pain is severe and the scalding troublesome, he applies 25 leeches to the perineum, and orders baths, cataplasms, &c., and in order to mitigate the painful erections, he gives

Camphor, 2 scruples;
Extract of opium, 8 grains; divide into 16 pills, two to be taken every evening.

When the disease has passed to the chronic stage, M. Ricord orders

Powdered cubebs, 4 ounces;
Peroxide of iron, 2 drachms; a powder to be taken every day; in addition, the patient is directed to inject, four times a day, a fluid composed of

Distilled water, 8 ounces;
Nitrate of silver, 2 grains.

Finally, when the running is of very long standing, and constitutes what is called an old gleet, he makes the patient inject the following solution,

Red wine, 6 ounces;
Tannin, 20 grains.

The regimen, during the acute stage, is mild and mucilaginous. Drinks are frequently administered.

---

*Kermes Mineral in Pneumonia.*—Dr. Lemarchand, of Le Mans, publishes several observations, in the Journal des Connaissances Medico-Chirurgicales for February, illustrative of the good effects of kermes mineral, in large doses, in conjunction with general and local bleeding, counter-irritation, &c., in the treatment of pneumonia. His cases show rapid amelioration of the symptoms, under the use of the remedy, which was not followed by either diarrhœa or vomiting.

---

*On the Medicinal Powers of the Caïnca, by Dr. Robredo.* Baron Langdorf, Russian consul-general at Rio Janeiro, first announced, a few years ago, the wonderful virtues of this plant in Europe. It is the *Chiococca racemosa* of Linnæus, and of the natural family *Rubiaceæ* of Jussieu, and is very abundant in the Antilles, the Floridas, and many parts of Brazil. Its root is composed of a delicate bark, which has a bitter and slightly acrid and astringent taste, and, when broken, offering a resinous aspect; and a woody centre, which does not partake of these properties. Messrs. Pelletier and Caventou have found that it contains a bitter principle, to which they have given the name of Caïncic acid, an oily matter of a green colour, another of a yellow colour, and a coloured viscous substance. From the experiments of Messrs. Caventou and François, it appears that the root, and especially the caïncic acid, are powerfully tonic; that they increase the secretion of urine in a very remarkable manner, and that they are slightly purgative.

Three cases are related by Dr. Robredo in confirmation of these opinions. In the first case, a bilious diarrhœa had reduced the strength of the patient, a female, extremely; the suppression of this diarrhœa being always followed by thirst, scanty urine, extensive œdema, and excessive prostration. A recurrence of the diarrhœa removed these symptoms, but exhausted rapidly the strength of the patient. The employment of caïnca was begun, when the dropsical symptoms were extreme and the urine scanty and sedimentous. A decoction of a drachm of the root in a pint of water was administered daily. The second day of its employment ten pints of clear urine were discharged, the stomach performing its functions regularly; and in five days a cure was effected, which the author thinks is radical, since, at the period of his writing, three months had elapsed without any disturbance of the patient's health, and her strength was quite restored.

In the second case, amenorrhœa had been treated by copious bloodletting, and anasarca was the consequence. The author having ascertained, by examination, that there was no disease of the uterus, administered caïnca. The ultimate effect was more slowly produced; fifteen days having been required for a cure, and the dose of the medicine (probably from deficient susceptibility of the patient,) having required increasing to a drachm and a half daily.

In the third case, that of a boy eight years old, there was extreme marasmus, œdema, costive bowels, and enlargement of the spleen; the whole the result of an ague of three months' duration. All the symptoms, the enlargement of the spleen excepted, were removed in eight days, by means of a decoction of half a drachm of the root daily; and, by appropriate remedies, the spleen was afterwards reduced to its natural condition.—*Brit. and For. Med. Review, from Jornal de la Academia de Medicina de Megico. Octubre*, 1836.

---

*Notice of the Bafureira of Cape de Verd, by Dr. B. A. Gomes.*—This plant is indigenous at Cape Verd, and is employed by the inhabitants of the Cape Verd Islands, and the coast of Africa, to increase the secretion of milk. The writer has been assured by persons whom he thinks worthy of credit, that, not only is it efficacious for this purpose in women who have been recently confined, but that it produces the secretion in virgins and persons of advanced life; so that infants have been nursed for a long time by females who, from their age and other circumstances, could not have furnished the secretion under the influence of any natural stimulant. Its mode of employment is by means of poultices, made of the green leaves, applied to the mammæ; or a strong decoction, with which the same parts and external organs of generation are washed. Sometimes such a decoction is taken internally, conjointly with its external application. The plant belongs to the family *Euphorbiaceæ* and the genus *Ricinus*. The writer is doubtful whether it is a variety of the *Ricinus communis*, or a distinct species. It has been raised in the garden of the Marine Hospital at Lisbon, from seeds sent from Cape Verd.—*Ib., from Jornal da Sociedade das Sciencias Medicas de Lisboa. Novembro*, 1836.

